# Sleep EEG Derived From Behind-the-Ear Electrodes (cEEGrid) Compared to Standard Polysomnography: A Proof of Concept Study

**DOI:** 10.3389/fnhum.2018.00452

**Published:** 2018-11-26

**Authors:** Annette Sterr, James K. Ebajemito, Kaare B. Mikkelsen, Maria A. Bonmati-Carrion, Nayantara Santhi, Ciro della Monica, Lucinda Grainger, Giuseppe Atzori, Victoria Revell, Stefan Debener, Derk-Jan Dijk, Maarten DeVos

**Affiliations:** ^1^School of Psychology, Faculty of Health and Medical Sciences, University of Surrey, Guilford, United Kingdom; ^2^Institute of Biomedical Engineering, University of Oxford, Oxford, United Kingdom; ^3^Surrey Sleep Research Centre, University of Surrey, Guildford, United Kingdom; ^4^Surrey Clinical Research Centre, Department of Psychology, University of Surrey, Guildford, Germany; ^5^Neuropsychology Lab, Department of Psychology, University of Oldenburg, Oldenburg, Germany; ^6^Cluster of Excellence Hearing, University of Oldenburg, Oldenburg, Germany

**Keywords:** electroencephalography, monitoring, sleep recording, home polysomnography, sleep stages, wake

## Abstract

Electroencephalography (EEG) recordings represent a vital component of the assessment of sleep physiology, but the methodology presently used is costly, intrusive to participants, and laborious in application. There is a recognized need to develop more easily applicable yet reliable EEG systems that allow unobtrusive long-term recording of sleep-wake EEG ideally away from the laboratory setting. cEEGrid is a recently developed flex-printed around-the-ear electrode array, which holds great potential for sleep-wake monitoring research. It is comfortable to wear, simple to apply, and minimally intrusive during sleep. Moreover, it can be combined with a smartphone-controlled miniaturized amplifier and is fully portable. Evaluation of cEEGrid as a motion-tolerant device is ongoing, but initial findings clearly indicate that it is very well suited for cognitive research. The present study aimed to explore the suitability of cEEGrid for sleep research, by testing whether cEEGrid data affords the signal quality and characteristics necessary for sleep stage scoring. In an accredited sleep laboratory, sleep data from cEEGrid and a standard PSG system were acquired simultaneously. Twenty participants were recorded for one extended nocturnal sleep opportunity. Fifteen data sets were scored manually. Sleep parameters relating to sleep maintenance and sleep architecture were then extracted and statistically assessed for signal quality and concordance. The findings suggest that the cEEGrid system is a viable and robust recording tool to capture sleep and wake EEG. Further research is needed to fully determine the suitability of cEEGrid for basic and applied research as well as sleep medicine.

## Introduction

Since the discovery of the sleep electroencephalogram (EEG) in humans (Loomis et al., 1937), physiological studies on sleep (polysomnography, PSG) and its disorders have been primarily conducted in specialized sleep laboratories, often within research settings. With the advancement of increasingly sophisticated measurements and methods, studying sleep in the home environment has become a feasible option. For example, a clinical review on six studies comparing home-PSG vs. lab-PSG for sleep disordered breathing diagnostics concludes that home-PSG is a reliable alternative to lab-PSG (Bruyneel and Ninane, 2014). With regards to sleep continuity, three studies report higher sleep efficiency and longer total sleep time for home compared to lab-based PSG (Portier et al., 2000; Iber et al., 2004; Bruyneel et al., 2011). However, others report the opposite (Fry et al., 1998) or no difference (Kingshott and Douglas, 2000; Campbell and Neill, 2011). Home-PSG studies further showed increased slow wave and rapid eye-movement sleep (REM), and lower sleep fragmentation (Kingshott and Douglas, 2000; Iber et al., 2004; Bruyneel et al., 2011), indicating that not only sleep maintenance but also sleep architecture might be better at home than in the lab. Further support for this notion is provided by Edinger’s studies (Edinger et al., 1997, 2001) which demonstrated differences in sleep continuity and sleep architecture indicative of better sleep at home in a within-subject paradigm of three nights of lab- and home PSG, respectively. However, whether these differences reflect genuinely better sleep at home or methodological differences is disputed.

Compared to the vast number of laboratory-based PSG studies, the number of home studies is minute. The findings summarized above highlights the need to study sleep and its disorders in the home environment. With recent technological advancements in transportable EEG systems, home-based PSG is becoming increasingly feasible. However, such studies remain difficult and costly because standard EEG systems require a trained technician for application. Moreover, the way standard EEG electrodes are mounted to the head is not conducive to natural sleep. The cEEGrid (Debener et al., 2015) is a flex-printed superthin adhesive strip with 10 embedded electrodes, shaped to fit neatly behind the ear. It has recently been developed and successfully applied in basic cognitive neuroscience research (Debener et al., 2015). Subsequent work has demonstrated that the data quality from cEEGrid affords reliable event-related potential components, such as the P300, N1, and other EEG markers of cognitive function (Bleichner et al., 2016; Kotz et al., 2016; Mirkovic et al., 2016; Pacharra et al., 2017); for review, see Bleichner and Debener (2017).

cEEGrid is particularly attractive for sleep recordings as it is easily applicable and holds the potential to be self-administered in the home environment. This makes prolonged monitoring of sleep EEG over several days affordable and feasible. The latter is important to monitor physiological sleep changes as they occur in a person’s life, for example in relation to physical or mental illness, or intervention over several days. Furthermore, the positioning of the electrode is minimally intrusive and thus, in principle, allows for natural sleep to be measured around the clock.

In the present study we examined the suitability of sleep data collection with cEEGrid combined with a miniaturized amplifier (Smarting, mBrainTrain, Serbia) and an off-the-shelf smartphone (Sony Z1) for sleep research. Specifically, we evaluated whether cEEGrid data affords the signal quality and characteristics necessary for sleep stage scoring. A secondary purpose of the study was to gain insight into the user friendliness of the set up for both participants and technicians. Sleep EEG was acquired simultaneously from a standard PSG system (SOMNOmedics Gmbh, Germany) and the cEEGrid system for one night. The study took place at the sleep laboratory of the Clinical Research Centre at the University of Surrey.

## Materials and Methods

### Participants

Twenty volunteers aged 34.9 ± 13.8 years (mean ± SD; 8 males) were recruited from the University of Surrey and the general public. Five data sets were lost due to technical problems with either the SomnoHD or the cEEGrid system (i.e., data loss, excessive artifacts, abortion of data collection by participant, user errors arising from the technical challenges to align the recordings, or a combination of these). The final sample comprised 15 participants (6 male), aged 35.3 ± 14.3 years [mean ± SD; range: 18–63] and a BMI of 24.1 ± 3.3 [mean ± SD; range 20.3–29.4]. They were mostly good sleepers [Pittsburgh Sleep Quality Index, PSQI (Buysse et al., 1989): mean: 2.93; SD: ±1.71; range: 0–6] and of intermediate chronotype [Morningness-Eveningness Questionnaire, MEQ (Horne and Ostberg, 1976): mean: 50.4, SD: ±12.87, range: 15–74].

Data collection took place in the sleep laboratory of the Surrey Clinical Research Centre (CRC) housed by the University of Surrey. The data collection was performed by trained staff. The protocol was approved by the University of Surrey Ethics committee. All participants gave written informed consent in compliance with the Declaration of Helsinki. All data obtained from the study were stored in accordance with the Data Protection Act (1998).

### Experimental Procedure

**FIGURE 1:**
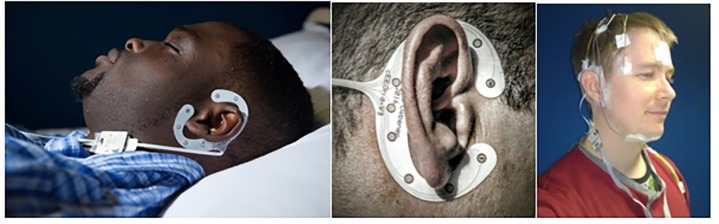
Data collection setup. **Left**: cEEGrid system set up (without PSG) in sleeping position. **Middle**: Close up of cEEGrid electrode. **Right**: Simultaneous montage of standard PSG and cEEGrid setup. Consent/permission was obtained from the individuals (JE and KBM) for the publication of this image.

Participants arrived at CRC by 17:00 h and left at approximately 11:00 h the following morning (lights off between 21.40 and 00.33; lights on between 7.23 and 10.38). Participants could turn the lights off when they wanted; bed time was habitual. Complying with centre’s standard operating procedures, admission procedures included a basic health check, breathalyzer test, urine sampling to test for drugs of abuse, and a pregnancy test for females. Afterward, participants were given three pre-programmed actiwatches (MW8 and AWL, CamNTech, United Kingdom; AX3 Activity Monitors, Axivity, United Kingdom) to be worn on the dominant hand (note that the actigraphy data is not presented in this paper). Subsequently participants were fitted with the electrodes and sensors of the SomnoHD system (SOMNOmedics Gmbh, Germany) and then the cEEGrid system. The setup is shown in Figure 1.

The SomnoHD is a wireless PSG system but also records locally to an SD card. It is approved and validated for sleep medicine. Sensor setup followed the CRC standard operating procedure and included six scalp electrodes (F3, F4, O1, O2, C3, and C4) referenced against the contralateral mastoid (A1 and A2) augmented with two ECG leads (electrocardiogram), two EOG electrodes (electrooculogram; outer canthi) and two EMG chin electrodes (electromyogram). Electrodes A1/A2 were positioned slightly more posterior than the standard mastoid position to make room for the cEEGrid strip. Electrode locations were measured and marked by hand using the international 10–20 system; head electrodes were mounted with Grass EC2 electrode cream (Natus Medical Inc., United States). All sensors were connected to the headbox attached to the person with a belt around the chest. The headbox was connected wirelessly to a recording unit placed at the back of the room. Data was sampled with 128 Hz.

**FIGURE 2:**
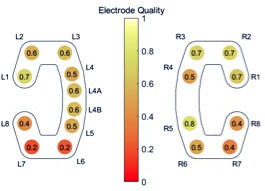
Overview of the reliability of each electrode, measured as the fraction of total recording time available after electrode rejection.

Once the electrodes and sensors for the SomnoHD system were fitted and worked (i.e., provided clear signals), one cEEGrid electrode strip with 10 electrodes each (TMSi, Netherlands) was fitted behind each ear using EEG conductive gel (Abralyt, Easycap, Germany) and double-sided adhesive stickers. The skin was cleaned with alcohol and abralyt before the adhesive sticker was attached. The electrodes from both cEEGrids were connected to a miniaturized 24-channels EEG amplifier equipped with a 3D gyroscope (SMARTING, mBrainTrain, Belgrade, Serbia). The signal was transmitted from the amplifier, mounted on the person via Bluetooth to a commercial mobile phone (Sony X1). The amplifier and the phone were placed in a small pocket that was velcro-taped to a soft adjustable belt worn around the waist. The amplifier characteristics comprised a sampling rate of 250 Hz, a resolution of 24 bits and a bandwidth from DC to 125 Hz. The phone was attached to a power-pack to guarantee full 12 h recordings. Impedances were kept <5 kΩ for SomnoHD and <20 kΩ for cEEGrid. In cases where an electrode within the cEEGrid strip could not be brought below the <20 kΩ threshold easily, it was switched off for the recording. This approach was chosen because fitting cEEGrid efficiently formed part of our feasibility assessment. Note that “losing” an electrode from the cEEGrid strip was acceptable because of its excellent spatial sampling. For further illustration, Figure 2 illustrates the reliability per electrode on group level, measured as the fraction of total recording time available after electrode rejection. Bio-calibrations were performed for both systems prior to lights out. The data streams from the two systems were aligned through artifact matching across the whole signal. This was done by finding the best fit between the traces in the cross correlation.

Data collection started after both systems were fitted. Participants were allowed to sleep *ad libitum* but were asked to stay in bed for 12 h. While in bed awake, participants were allowed to read or listen to music; they could switch on when they wanted to. This extended sleep opportunity approach was chosen to ensure that the recordings contained not only sleep but also a substantial amount of wake EEG.

### Data and Statistical Analysis

The data files from SomnoHD and cEEGrid were converted to EDF format and randomized for blinding before being read into the software DOMINO^®^ (SOMNOmedics Gmbh, Germany) for visualization and scoring. Each dataset was scored manually by a trained and highly experienced registered polysomnographic technologist following the guidelines of the American Academy of Sleep Science (AASM) to obtain the hypnograms. All recordings, PSG and cEEGrid, were independently scored by two experienced sleep technologists, in accordance with the American Association for Sleep Medicine (AASM) guidelines. For the PSG-SomnoHD recordings, standard derivations were used to score the records. cEEGrid recordings were scored as they were displayed on the screen, i.e., single channels derivations, e.g., L3. Continuous visual inspection was used by the scorer(s) to determine the channel(s) that best fitted the scoring criteria. To eliminate bias, the cEEGrid recordings were anonymized prior to scoring to ensure that the scorers could not tell whether they were scoring the records of the same subject.

From these scores sleep parameters (see Table 1) were extracted with SAS. For concordance analysis all datasets were scored by two persons. The statistical analysis of the sleep parameters was conducted with the data from scorer one.

**Table 1:** Abbreviations and definition of sleep parameters.

Abbreviation	Definition
EUS (count)	Epochs of un-scored sleep: epochs not classified as a sleep stage, wake or artifact
DUR _W (min)	Stage W duration: time spend as wake
DUR_REM (min)	Duration of REM: time spend in REM sleep
DUR_NREM	Duration of NREM sleep: time spend in N1, N2, and N3 combined
DUR_N1 (min)	Stage 1 duration: time spent in stage N1
DUR_N2 (min)	Stage 2 duration: time spent in stage N2
DUR_N3 (min)	Stage 3 duration: time spent in stage N3
%N1	Percent N1: percentage amount of TST spend in stage 1 sleep
%N2	Percent N2: percentage amount of TST spend in stage 2 sleep
%N3	Percent N3: percentage amount of TST spend in stage 3 sleep
TRT (min)	Total recording time: time for which data was recorded and analyzed (note: this includes periods of sleep and quiet rest).
TST (min)	Total sleep time: time scored as NREM or REM excluding epochs classified as “Unsure” or “Wake”
SPT (min)	Sleep period time: total time scored as NREM, REM, or WAKE occurring from sleep onset to final wake
SE (%)	Sleep efficiency: percentage of TST against TRT
WASOSP (min)	Wake after sleep onset: time in minutes scored as wake from sleep onset (SOL) to final awakening.
SOL (min)	Sleep onset latency time in minutes from start of recording to the first epoch of NREM or REM
REM_L (min)	REM sleep latency, time in minutes from SOL to the first epoch of REM
LPS (min)	Latency to persistent sleep: time from start of recording to the first consecutive 20 epochs of NREM or REM

For validating that cEEGrid captures overall neurophysiology, relative alpha and delta power was computed for NREM (non-REM) periods and quality assessed through correlations. To assess the quality of manual sleep staging for cEEGrid, concordance rates between cEEGrid and SomnoHD were calculated as the % of artifact-free epochs with matching classification. Inter-rater concordances between scorers were further calculated for both systems.

As an indirect measure of coherence between the sleep staging of SomnoHD and cEEGrid data, Pearson correlations were calculated. To assess the overall similarities of the histograms obtained from the SomnoHD and cEEGrid, Cohen’s kappa was calculated for all sleep stages as well as the discrimination between restful wake and sleep. Limits of Agreement were calculated for each parameter according to the Bland Altman method (Bland and Altman, 1986), and tested for significant deviation from zero with paired-samples *t*-tests. Calculations were performed with Excel v16 and SPSS v25.

## Results

The mean recording times for the two systems were 11:47 for SomnoHD and 11:10 for cEEGrid. The analysis period used for sleep scoring of both systems averaged 11:25 with a minimum 09:25 and a maximum of 11:58 except for one participant whose cEEGrid recording was truncated after 04:20.

**FIGURE 3:**
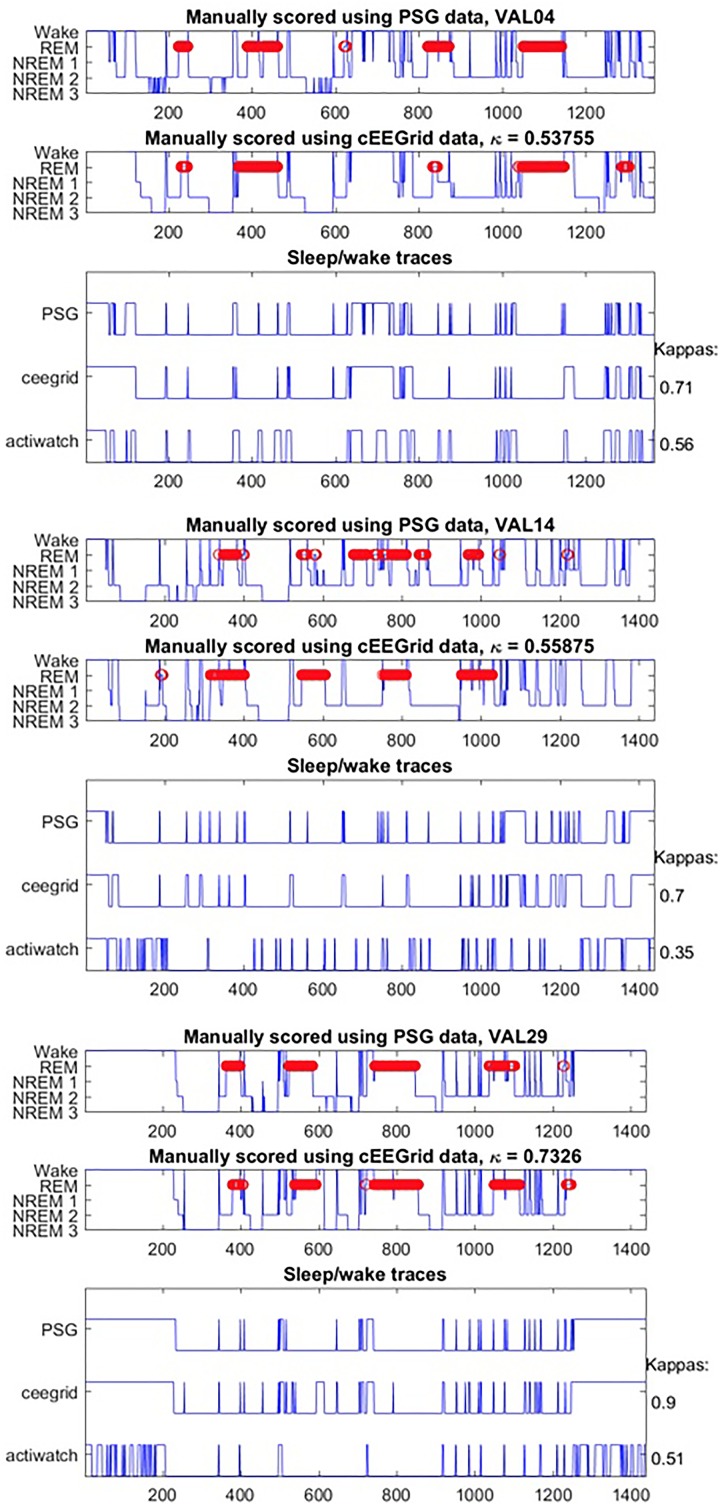
Hypnograms from three participants.

**FIGURE 4:**
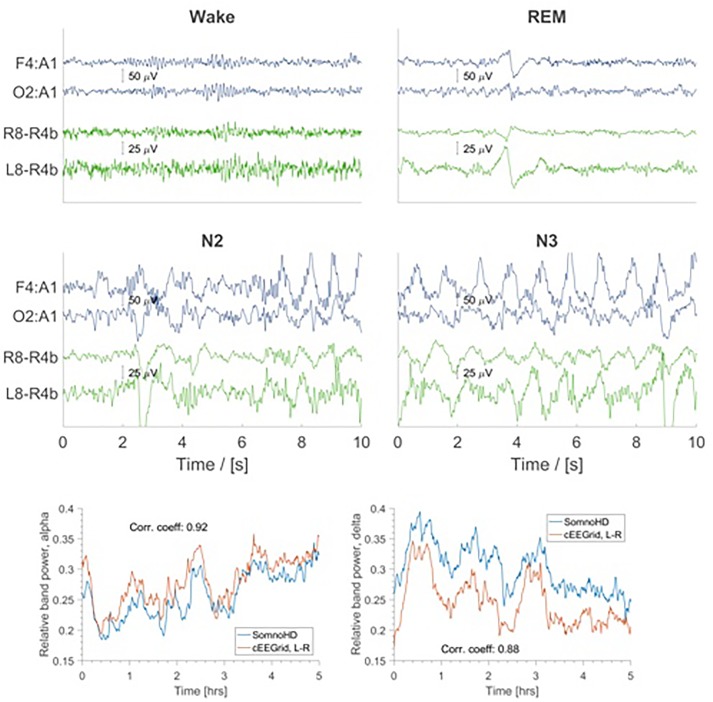
Top four panels: EEG traces for SomnoHD (blue) and cEEGrid (green). Bottom panel: Average relative power in the alpha (8–16 Hz) and delta (1–4 Hz) frequency bands depicted for SomnoHD and cEEGrid. Not all subjects had 5 h worth of NREM recording, meaning that the number of subjects included in the average decreases with time.

Generally, data collection was well tolerated and no adverse effects were reported for cEEGrid. The signal quality was comparable for both systems throughout the recording period; however, we note that the overall signal strength was lower for cEEGrid than for SomnoHD. Exemplary hypnograms and traces for different sleep stages are presented in Figures 3 and 4, respectively. Mean values for the stages are summarized in Table 2.

**Table 2:** Means, standard deviation, and range for sleep parameters.

Parameter	SomnoSD	cEEGrid	Difference
EUS (count)	1.1 ± 1.8 [0.0-7.0]	11.9 ± 36.0 [0.0-140.0]	10.8 epochs (=5.4 min)
DUR_W (min)	207.2 ± 78.0 [65.5-313.5]	229.8 ± 145.4 [39.5-632]	22.6
DUR_REM (min)	81.0 ± 47.8 [0.0-135.5]	68.4 ± 50.8 [0.0-139.5]	12.6
DUR_NREM (min)	327.4 ± 102.1 [121.0-495.0]	306.6 ± 110.7 [76.5-442.5]	20.8
DUR_N1 (min)	45.3 ± 31.4 [3.5 ±113.0]	46.3 ± 32.6 [10.5-124.5]	-0.9
DUR_N2 (min)	210.7 ± 83.7 [37.5-326.0]	165.4 ± 78.1 [37.0-278.0]	45.2
DUR_N3 (min)	71.4 ± 22.5 [8.5-98.5]	94.9 ± 39.5 [5.5-151.5]	23.5
%N1	10.8 ± 6.7 [0.9-26.0]	14.1 ± 11.1 [4.9-44.4]	3.3
%N2	50.6 ± 9.0 [31.0-66.9]	44.2 ± 8.9 [24.2-54.3]	6.4
%N3	20.9 ± 13.7 [2.1-60.7]	26.3 ± 11.6 [7.2-45.8]	5.4
TRT (min)	616.6 ± 168.1 [187.0-719.0]	616.6 ± 168.1 [187-719]	0.0
TST (min)	408.3 ± 137.2 [121.0-598.0]	375.0 ± 151.3 [76.5-582]	33.4
SPT (min)	496.1 ± 163.4 [133.5-676.5]	479.3 ± 184.8 [116.5-692]	16.7
SE (%)	65.4 ± 10.2 [49.6-86.2]	62.1 ± 18.4 [10.8-84.7]	3.3
WASOSP (min)	87.7 ± 76.0 [5.0-242.5]	104.4 ± 76.9 [31.0-273.0]	-16.6
SOL (min)	90.5 ± 70.3 [4.0-276.0]	46.6 ± 44.9 [4.9-44.4]	43.8
REM_L (min)	100.3 ± 38.1 [56.5-160.5]	155.1 ± 87.5 [42.0-351.5]	-54.8
LPS (min)	97.1 ± 66.0 [10.0-276.0]	67.7 ± 42.3 [10.5-158]	29.4

*Note that the sleep parameters were based on the data from scorer one.*

On group level the kappa for all sleep stages combined was moderate according to the classification by Landis and Koch (1977) [0.42 ± 0.21; (moderate range: 0.41–0.6)]. On an individual level the kappas indicated slight agreement [0–0.20] in three participants, fair agreement [0.21–0.40] in three participants, moderate agreement [0.41–0.60] in seven participants, and substantial agreement [0.61–0.80] in two participants.

For the discrimination of quiet wake vs. sleep, the agreement was better than for sleep stages, with a group level kappa of 0.55 ± 0.24, with agreement being classified as slight in one participant, fair in three participants [0.21–0.40], moderate in six participants [0.41–0.60], substantial in four participants [0.61–0.80], and almost perfect in 1 participant [0.81–1].

The Bland Altman statistics, provided in Table 3, suggest good agreement for 9 out of the 13 sleep parameters. However, significant disagreement between the systems were found for N2, N3, and SOL; a trend was observed for REM_L. Pearson correlations by and large confirmed this observation with significant correlations between SomnoHD and cEEGrid scorings for 10 out of the 13 sleep variables and a trend for WASOSP (Table 4). The correlation for sleep efficiency was weak (*r* = 0.36), and <0.1 for REM latency.

**Table 3:** Bland Altman statistics for sleep parameters obtained from scorer 1.

Sleep parameter	Mean ± standard deviation	*t*-Test (two tailed)
DUR_W	-22.6 ± 102.3	*t*_(14)_ = -0.85; *p* = 0.41
DUR_REM	12.6 ± 45.0	*t*_(14)_ = 1.09; *p* = 0.30
DUR_NREM	20.8 ± 87.0	*t*_(14)_ = 0.93; *p* = 0.37
DUR_N1	0.93 ± 27.1	*t*_(14)_ = -0.13; *p* = 0.90
DUR_N2^∗^	45.2 ± 77.1	*t*_(14)_ = 2.27; *p* = 0.04
DUR_N3^∗^	-23.5 ± 30.8	*t*_(14)_ = -2.95; *p* = 0.01
TRT	0.00 ± 0.00	-
TST	33.4 ± 103.7	*t*_(14)_ = 1.25; *p* = 0.23
SPT	16.7 ± 158.2	*t*_(14)_ = 0.41; *p* = 0.69
WASOSP	16.6 ± 76.5	*t*_(14)_ = -0.84; *p* = 0.41
SOL^∗^	43.8 ± 58.6	*t*_(14)_ = 2.90; *p* = 0.01
REM_L	-54.8 ± 98.2	*t*_(14)_ = -2.16; *p* = 0.05
LPS	29.4 ± 48.6	*t*_(14)_ = 2.34; *p* = 0.40

*^∗^Indicates significant deviation from zero. Bland Altman plots are presented in the Supplementary Materials.*

**Table 4:** Pearson correlations between SomnoHD and cEEGrid for sleep parameters obtained from scorer 1.

Variable	*r*_(13)_	*p*-Value
DUR_W (min)^∗∗^	0.74	0.01
DUR_REM (min)^∗^	0.59	0.02
DUR_NREM (min)^∗∗^	0.67	0.01
DUR_N1 (min)^∗∗^	0.64	0.01
DUR_N2 (min)^∗^	0.55	0.05
DUR_N3 (min)^∗^	0.63	0.05
TST (min)^∗∗^	0.76	0.01
SPT (min)^∗^	0.60	0.02
SE (%)	0.36	0.18
WASOSP (min)	0.50	0.06
SOL (min)^∗^	0.56	0.03
REM_L (min)	0.08	0.77
LPS (min)^∗∗^	0.68	0.01

*^∗∗^Indicates p < 0.01; ^∗^indicates p < 0.05.*

Epoch by epoch concordances are presented in Table 5. Agreement between SomnoHD and cEEGrid data obtained from the same rater was 58.5%. Inter-rater concordances were very good for both systems with 97.3% agreement for SomnoHD and 99.3% for cEEGrid.

**Table 5:** Epoch-to-epoch concordance (%) for manual scoring.

	cEEGrid–SomnoHD	ICC SomnoHD	ICC cEEGrid
Mean	58.5%	97.3%	99.3%
Standard deviation	13.7	4.8	0.5
Minimum	28.2	84.7	97.9
Maximum	80.1	99.8	100.0

*Column 2: concordance rates between cEEGrid and SomnoHD data obtained by one scorer; columns 3 and 4: inter-rater concordances for SomnoHD and cEEGrid, respectively.*

## Discussion

The present study compared sleep EEG recorded with a standard PSG montage with the EEG obtained from an electrode strip mounted behind the ear recorded with a miniaturized wireless amplifier on a smartphone. The principal aim of the study was to explore whether cEEGrid in combination with a smart phone is a suitable tool for sleep research, and to provide initial evidence to support further development of the cEEGrid principle for self-administration. This general aim was supported by our findings and observations. The cEEGrid system was easy and fast to set up, and, despite the experimental nature of the system, robust against operator errors. It required only one person and a small amount of training to complete the whole setup within 20 min. For SomnoHD, electrode preparation took approximately 45 min when conducted by one well trained sleep technician. In addition, the study demonstrates that the combination of cEEGrid with a Smarting amplifier, mobile phone, and power pack, can be worn during sleep without major disturbance. It is robust enough to afford prolonged recordings (>12 h) and with four systems acquiring data in neighboring rooms simultaneously, i.e., without interference between systems operating in close proximity and/or other wireless data transmission devices. The latter is an important point not only for studies in clinical settings, where recordings may be taken from patients sleeping in multi-bed units and the presence of other electronic equipment, but also for field studies examining the effects of sleeping with a partner within the same bed.

The present findings indicate that cEEGrid can capture the sleep characteristics indicative of sleep continuity and sleep architecture. Moreover, the data confirms single case evidence (Bleichner and Debener, 2017) that the signal quality from cEEGrid was solid. This was evident in the frequency analysis which showed a similar change of frequency composition throughout the night for standard PSG and cEEGrid as evidenced in Figure 4. Moreover, as illustrated in Figure 2, most electrodes were stable throughout the recording. Importantly, because only the three best electrodes are needed for the derivations used for the EEG element of sleep stage scoring, not all electrodes necessarily need to work perfectly. This gives cEEGrid greater resilience against single electrode failure compared to systems with standard head electrodes. Taken together the findings from the present study suggest that cEEGrid warrants further exploration as a novel tool for sleep studies. In our view, it has the potential to be developed into a sleep monitoring tool that lends itself to self-administration in clinical and home environments, and as such, may provide a good tool to study natural sleep in many settings. However, further studies are clearly necessary before the latter can be affirmed.

The cEEGrid system tested in the present study seems to be well suited for sleep research. However, it is important to stress that the present study is only the first step to demonstrate that the extraction of sleep stages based on AASM criteria is possible. However, better AASM concordance between the two systems most likely requires adjustments of amplitude criteria for cEEGrid recordings. Importantly, our results do not provide any information with regards to the detection of organic sleep disorders, such as sleep disordered breathing, narcolepsy, and periodic limb movement syndrome. Moreover, the location of the electrode array poses challenges for the investigation of local sleep. The findings from the present study therefore not only need to be replicated but expanded in scope, and examine adaptations to the system to make it suitable for studies in sleep medicine.

Another area that requires further exploration regards the recording of eye movements. Thus, the typical PSG set up includes electrodes specifically placed around the eye to detect eye movements with maximal sensitivity. These eye movements are very important for manual sleep staging, in particular with regards to the discrimination of REM and non-REM sleep. cEEGrid, however, does not have these specific eye movement channels. The inter-rater concordance rates were excellent for both systems (97 and 99%, respectively). However, the concordance between systems was low (58%). and less good than gold standard requirements. The high inter-rater concordance for cEEGrid clearly suggests that AASM -trained scorers apply the staging criteria in a very consistent and reliable manner. However, the low between-systems concordance rate suggests a bias. The most likely source of this bias lies in the absence of specific derivations to capture eye movements. In theory, the signal of eye movements is fully represented in the signal picked up by cEEGrid. While not easily visible by eye initially, scorers may very well learn to “read” eye movements with training. Advanced methods implemented in autoscoring algorithms are also likely to detect stage-specific eye-movement characteristics (features). This raises the possibility that cEEGrid data may benefit from automated scoring algorithms that include feature detection and machine learning. Future research will examine this question.

The present study recorded data from 20 participants but five data sets were lost to technical problems and excessive artifacts. A data loss of 25% is severe and, at first glance, seems to question the robustness of the cEEGrid system. However, the data loss needs to be contextualized by the complexities involved in the simultaneous application of two different EEG systems. In addition, the cEEGrid set-up was used for the first time in a full sleep study and contained a number of in-house solutions that increased the demand on the experimenters even further. This led to a number of data sets being lost simply to human error with one or the other system. Out of the five lost datasets, two could be accounted specifically to cEEGrid. In one case the recording simply stopped for unexplained reasons and could not be fixed there and then. In the other case, the participant tried to put the Smarting software into viewer mode to see his EEG during the recording. By doing so the data collection was inadvertently terminated. The latter incidence highlights the need to develop better safeguards for adverse user interference before cEEGrid studies with minimally assistive self-administration are feasible on a larger scale. Moreover, prolonged wear of cEEGrid can cause discomfort if the device touches the pinnae of the ear. This was observed by other labs using cEEGrid as well as in the present study. Further advancement of cEEGrid should therefore include different cEEGrid sizes and the usage of softer materials for the adhesive strip on which the actual electrodes are printed.

Technological advancements for brain-based sleep research have had a lot of interest in recent years, and several studies with new/alternative sleep EEG systems have been published. For example, Younes et al. (2017) compared a head mounted wireless system (Prodigy) that relies on two channels obtained from the forehead, as well as the outer canthi, the chin and the mastoid with standard head-mounted EEG in 57 individuals with various sleep disorders. The study found good to excellent inter class correlations between manually scored PSG and autoscored Prodigy data. Using a similar type of approach with a multichannel frontopolar EEG device (Sleep Profiler), Levendowski et al. (2017) found good inter-rater reliability (73.1%) between automated scoring of Sleep profiler data and manually scored PSG. This demonstrates that reliable sleep EEG can be recorded with reduced electrode set ups that are more comfortable for participants to sleep with. Moreover, because these systems use forehead electrode positions, application of AASM criteria achieves good results. However, because of the nature of the electrode location, these systems are still relatively intrusive. This makes a system like cEEGrid, which is hardly visible, more useful in situations where prolonged sleep-wake recordings are required and data collection encompasses times when participants are not lying in bed. But clearly, the signal characteristics of cEEGrid pose challenges to manual scoring without specific training. This is evident in the weak correlations between cEEGrid and PSG scoring for sleep efficiency, sleep onset latency and REM latency and the low between-system concordance rate of 58%.

Much more research has to be conducted to determine the best way to reliably score sleep and wake data from cEEGrid electrodes if scoring is conducted by hand. The latter may very well include slight amendments to AASM criteria, as these rules are made for standardized scalp positions. Another way forward may lie in the application of machine-based learning algorithms that are able to extract features and patterns embedded in the signal but not necessarily distinguishable with the naked eye. This may make sleep data acquired with cEEGrid particularly suitable for autoscoring.

## Conclusion

cEEGrid represents a feasible and robust way of acquiring sleep EEG. Further advancement of the technology is necessary to allow self-administered application in the field. As pointed out in a recent review (Medic et al., 2017) sleep disruption has substantive short- and long-term consequences, and sleep EEG recordings therefore need to become cheaper and more accessible, yet scientifically sound, to determine the public health impact of these consequences. Our data suggests that cEEGrid fulfills these characteristics and hence has the potential to be developed into a tool that delivers population-based PSG availability at reasonable cost.

## Author Contributions

AS, MD, D-JD, and SD contributed in protocol and manuscript. JE, MABC, NS, VR, D-JD, and AS contributed in ethics. KM, NS, MD, and AS contributed in data analysis and statistics. GA, CdM, and LG contributed in sleep staging. JE, MABC, VR, and MD collected the data.

## Conflict of Interest Statement

JE, MABC, NS, VR, SD, D-JD, and AS declare no conflicts of interest, including any involvement in organizations with financial interest in the subject matter of the paper. KM and MD received a grant from Circadian Therapeutics to perform this study. MD is a founding member of Circadian Therapeutics. The remaining authors declare that the research was conducted in the absence of any commercial or financial relationships that could be construed as a potential conflict of interest.
