# Supplementary figures and images for: Sleep EEG Derived From Behind-the-Ear Electrodes (cEEGrid) Compared to Standard Polysomnography: A Proof of Concept Study
Source: Front Hum Neurosci. 2018 Nov 26;12:452. doi: 10.3389/fnhum.2018.00452 (PMC6276915; doi:10.3389/fnhum.2018.00452)

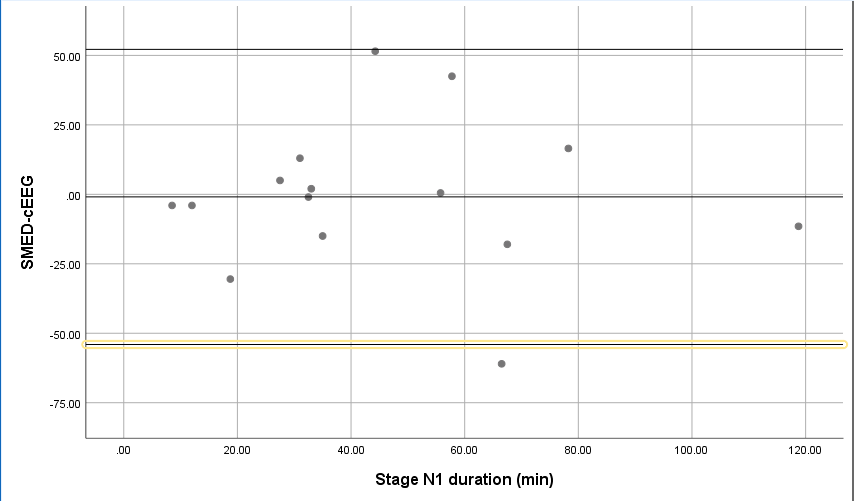

Supplement: Supplementary file 1 [file Presentation_1.zip › Image 1.PNG]

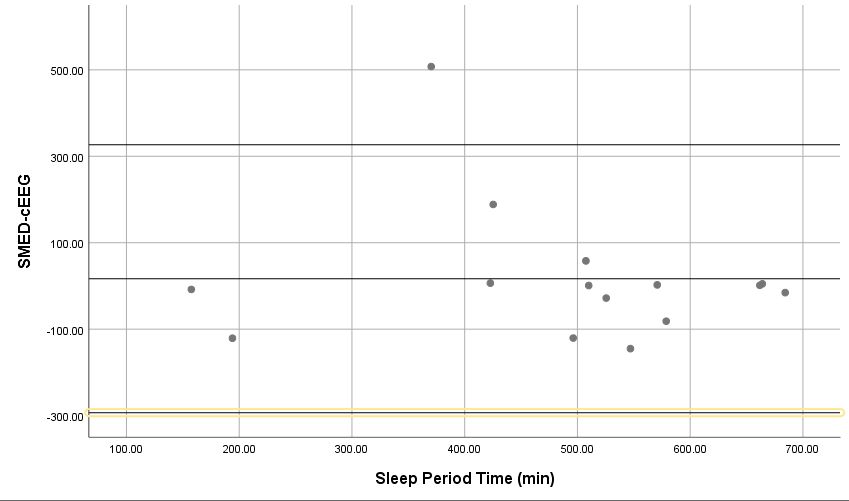

Supplement: Supplementary file 1 [file Presentation_1.zip › Image 10.PNG]

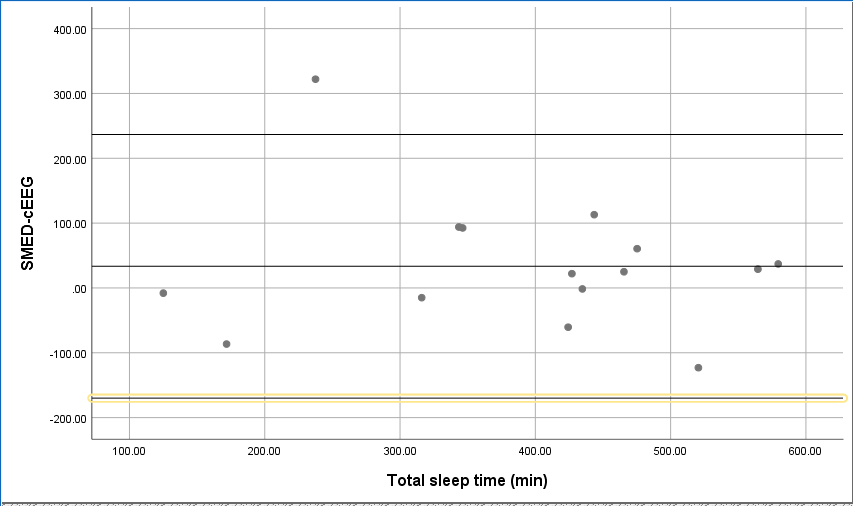

Supplement: Supplementary file 1 [file Presentation_1.zip › Image 11.PNG]

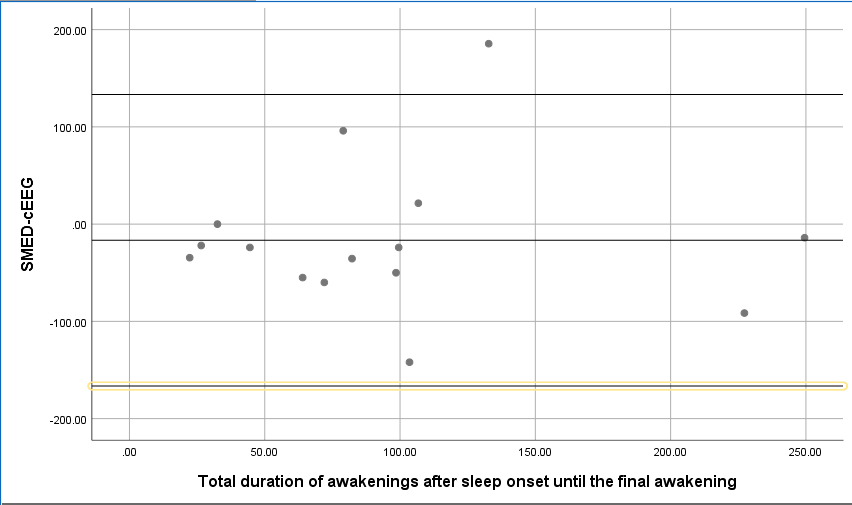

Supplement: Supplementary file 1 [file Presentation_1.zip › Image 12.PNG]

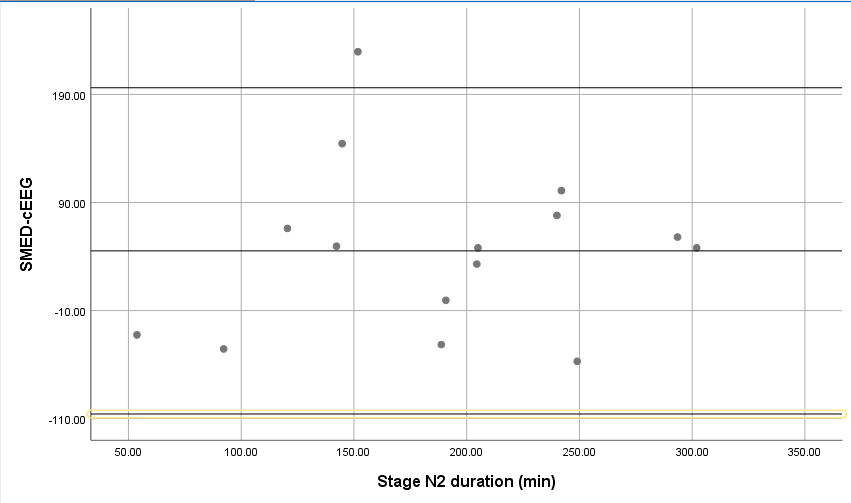

Supplement: Supplementary file 1 [file Presentation_1.zip › Image 2.PNG]

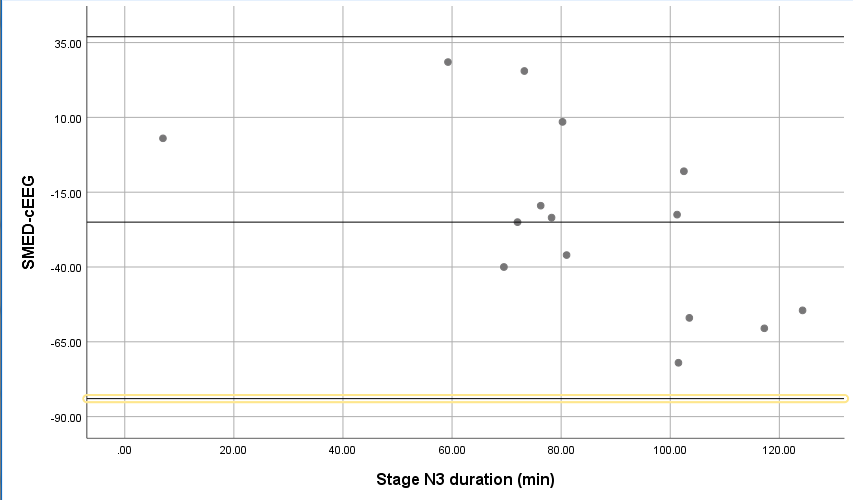

Supplement: Supplementary file 1 [file Presentation_1.zip › Image 3.PNG]

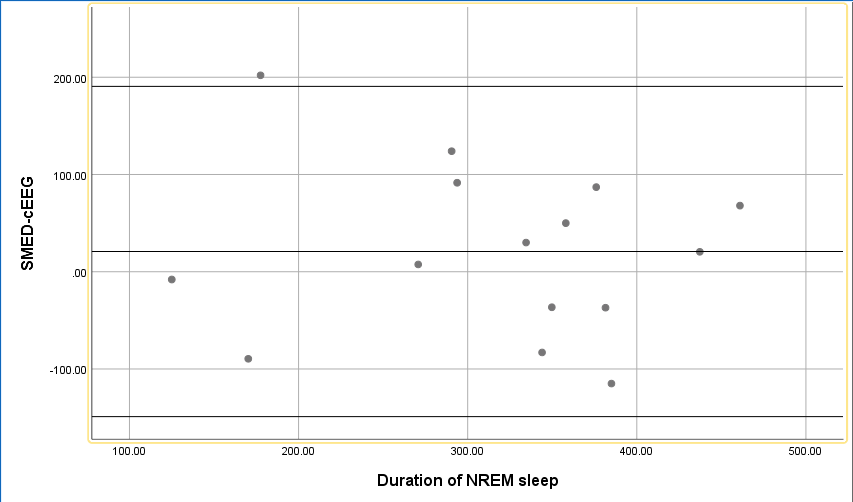

Supplement: Supplementary file 1 [file Presentation_1.zip › Image 4.PNG]

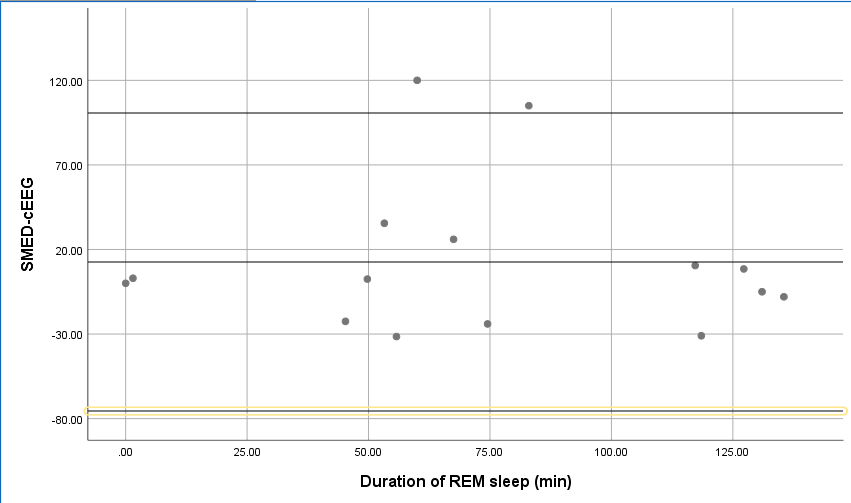

Supplement: Supplementary file 1 [file Presentation_1.zip › Image 5.PNG]

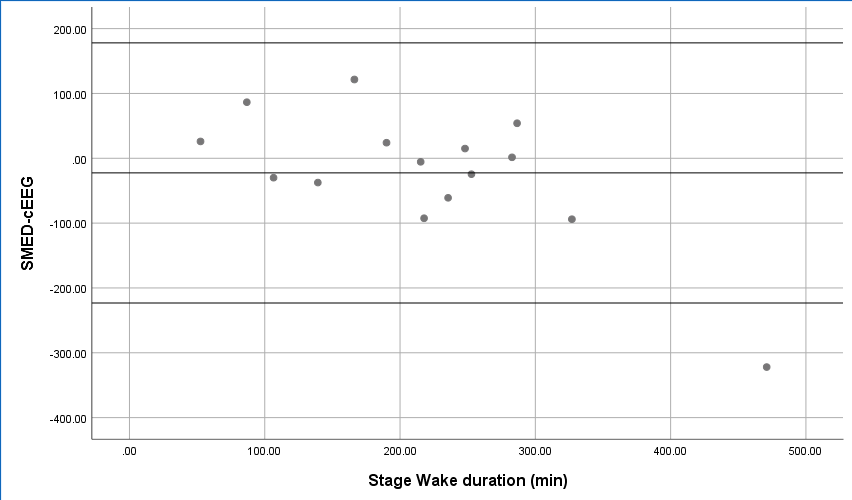

Supplement: Supplementary file 1 [file Presentation_1.zip › Image 6.PNG]

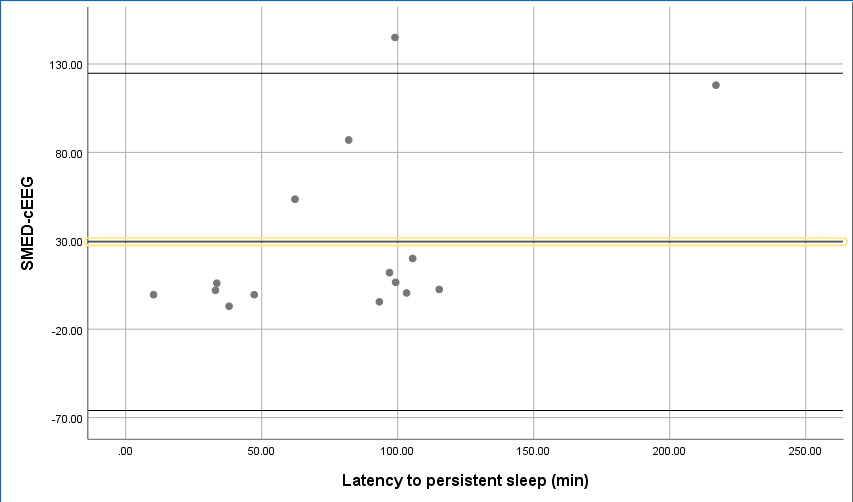

Supplement: Supplementary file 1 [file Presentation_1.zip › Image 7.PNG]

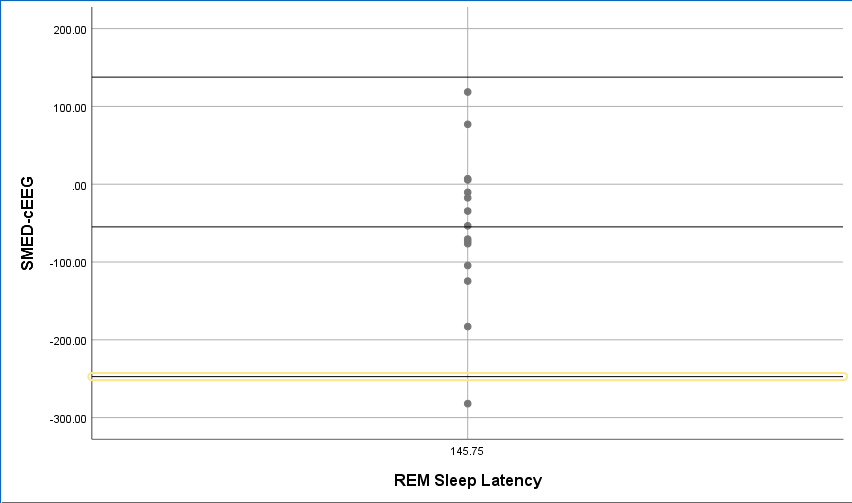

Supplement: Supplementary file 1 [file Presentation_1.zip › Image 8.PNG]

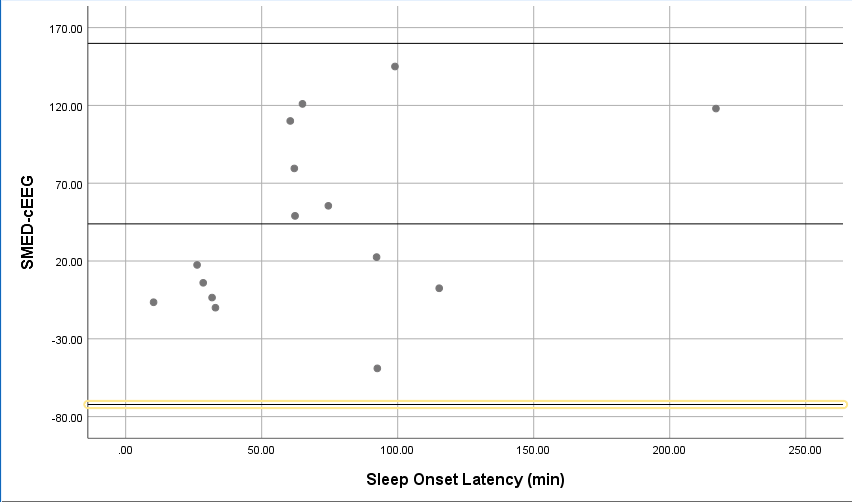

Supplement: Supplementary file 1 [file Presentation_1.zip › Image 9.PNG]
